# Pathogen diversity of the neglected winter tick *Alloceraea inermis* in Hungary

**DOI:** 10.1016/j.crpvbd.2026.100389

**Published:** 2026-05-20

**Authors:** Éva S. Szabó, Margarida Ruivo, Domonkos Adorján Köves, Máté Miklós, Flóra Kulin, Michiel Wijnveld, Gábor Földvári

**Affiliations:** a Institute of Evolution, HUN-REN Centre for Ecological Research, Konkoly-Thege Miklós út 29-33, Budapest, 1121, Hungary; b Centre for Eco-Epidemiology, National Laboratory for Health Security, Konkoly-Thege Miklós út 29-33, Budapest, 1121, Hungary; c Doctoral School of Biology, Institute of Biology, Eötvös Loránd University, Pázmány Péter sétány 1/c, Budapest, 1117, Hungary; d Institute for Hygiene and Applied Immunology, Center for Pathophysiology, Infectiology and Immunology, Medical University of Vienna, Kinderspitalgasse 15, Vienna, 1090, Austria; e Institute of Epidemiology and Scientific Research, Hungarian Defence Forces Medical Centre, Róbert Károly Boulevard 44, Budapest, 1134, Hungary

**Keywords:** Winter tick, *Alloceraea inermis*, *Haemaphysalis inermis*, Neglected species, *Rickettsia*, DAMA protocol, Reverse line blot

## Abstract

Ticks are important vectors of pathogens affecting human and animal health, making the identification of tick-borne agents essential. This study focused on the understudied winter tick, *Alloceraea inermis* (syn. *Haemaphysalis inermis*), which is widely distributed across Eurasia and, in some regions, appears to be increasing in occurrence. This species may play an important role in maintaining and potentially transmitting pathogens among its animal hosts. Although *A. inermis* rarely feeds on humans, occasional human parasitism does occur. Over two years, a total of 208 *A. inermis* ticks (123 females and 85 males) were collected from vegetation in a deciduous forest in Hungary during its autumn-winter activity period. Pathogen screening was performed using a reverse line blot hybridization assay targeting members of the genera *Anaplasma*, *Babesia*, *Borrelia*, *Ehrlichia*, *Rickettsia*, and *Theileria*. Species of *Rickettsia* were the most prevalent pathogens, with *Rickettsia helvetica* detected in 25.5% (53/208) of the samples, along with evidence for an uncharacterized *Rickettsia* species. Species of *Babesia* were the second most common pathogen group, with *Babesia microti* identified in 19.2% (40/208) of the ticks. Species of *Borrelia* were detected in 16.3% (34/208) of the samples, and “*Candidatus* Neoehrlichia mikurensis” was present in 14.9% (32/208) of the samples. The detected pathogens were more diverse and more frequent than expected, in some cases even surpassing prevalences in *Ixodes ricinus* observed in previous studies. In the future, we aim to clarify the vector role of *A. inermis* and assess associated health risks.

## Highlights

• A total of 208 adult winter ticks, *Alloceraea inermis*, were tested for multiple tick-borne bacteria and protozoans.
• Most frequently detected pathogens were *Rickettsia*, *Babesia*, *Borrelia* spp. and “*Candidatus* Neoehrlichia mikurensis”.
• Pathogen diversity and prevalence was higher than expected, some even surpassing those in *Ixodes ricinus.*
• Misconceptions about tick inactivity during winter may increase exposure to adult *A. inermis* ticks.

## Graphical abstract

**Figure:**
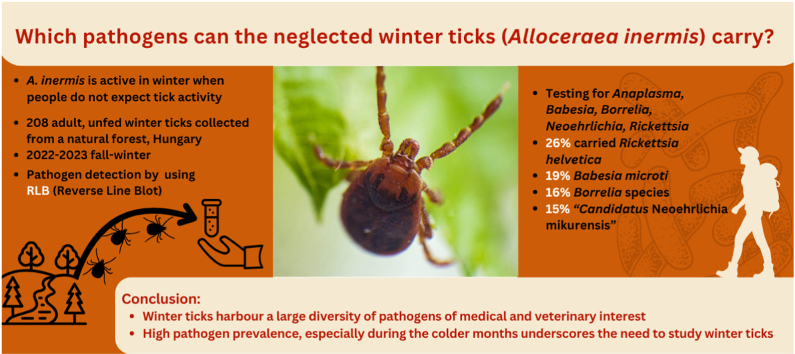

## 1 Introduction

*Alloceraea inermis*, originally named *Haemaphysalis inermis* and reclassified under its current status by Kelava et al. (2024), is a unique tick species with distinctive traits and behaviors that set it apart from other ticks (Kelava et al., 2024). Commonly referred to as the winter tick, this species is notable for the activity of its adult stage during the colder months (typically from November to February in Central Europe). This species has been reported across the Middle East, South Asia, and parts of Europe. In some European countries, such as Hungary and Spain, it has recently been found at relatively high abundance, suggesting a possible expansion of its range (Otranto et al., 2017). A significant feature is its unique seasonal activity in adults and its rapid feeding cycle in immature stages. While adult females can remain attached for weeks, laboratory studies show that larvae and nymphs complete feeding within a few hours, minimizing the time spent attached to their host (Nuttall and Warburton, 1915). In contrast, the feeding period of the closely related *Haemaphysalis punctata* and *Haemaphysalis concinna* nymphs and larvae is approximately 3–4 days long (Nosek, 1973).

Adults of *Alloceraea inermis* seek hosts during the colder months and often withstand rain and snowfall on vegetation. This activity pattern allows adults to exploit a seasonal niche not used by other tick species, which are typically less active or have a diapause in winter. In contrast, *A. inermis* larvae and nymphs are active from April to August, favoring the warmer months to quest for hosts in vegetation (Nosek et al., 1967; Santos-Silva et al., 2011; Otranto et al., 2017).

Ticks of this species feed on a wide range of larger mammals and livestock, including sheep, goats, cattle, water buffalo, red foxes, horses, and dogs. There are also published reports from humans. Larvae and nymphs prefer small mammals to birds or lizards (Bursali et al., 2012; Otranto et al., 2017).

Experimental evidence demonstrated the vectorial capacity of *A. inermis* for tick-borne encephalitis virus (TBEV). In Slovakia, this species has been implicated in the potential spread of TBEV (Gresíková and Nosek, 1966). In previous studies, *A. inermis* nymphs transmitted TBEV after feeding as larvae on virus-infected mice, confirming the tick’s potential role as a TBEV vector under controlled conditions (Gresíková and Nosek, 1966; Nosek et al., 1981).

Recent studies suggest that *A. inermis* may play a role in transmitting several pathogens, including various rickettsial species (Ouarti et al., 2022). In northern Spain, *A. inermis* has been linked to the transmission of *Rickettsia aeschlimannii*, an agent of tick-borne spotted fever, a disease known to cause fever, headache, and rash in humans (Portillo et al., 2008). Additionally, this tick species was identified as a potential vector for *Rickettsia helvetica*, a pathogenic *Rickettsia* species associated with febrile illness and, in some cases, neurological symptoms, such as meningitis in humans (Nilsson et al., 2010). In a study conducted in Hungary, some specimens tested positive for a novel genotype, “*Candidatus* Rickettsia hungarica”, which shares 95.8% similarity in the citrate synthase (*gltA*) gene with known rickettsiae (Hornok et al., 2010).

Furthermore, studies in Spain and Hungary have implicated *A. inermis* as a potential vector for *Babesia bigemina*, a protozoan parasite that causes bovine babesiosis, an economically significant disease in livestock (García-Sanmartín et al., 2008). Additionally, *A. inermis* has been found to harbour several other pathogens, including *Anaplasma bovis*, *Babesia crassa*, and *Borrelia miyamotoi*, showing its potential role in the ecology of multiple infectious agents (Hornok et al., 2015; Pazhoom et al., 2016; Heglasová et al., 2020).

While these findings indicate an association between *A. inermis* and various pathogens, its role as a vector under natural conditions remains unclear. The tick’s ability to serve as a competent vector may depend on specific environmental conditions, like host availability, climate, and interactions with other tick species. Nevertheless, the presence of *A. inermis* in multiple regions, along with its association with diverse pathogens, shows the need for continued research into its vector potential and public health implications, particularly in areas experiencing shifts in tick populations and pathogen prevalence due to climate change.

Forests, parks, and hiking trails near urban areas, as well as national parks, have become increasingly popular spots for recreation. Many people frequent these natural areas for leisure, and some professions (e.g. forestry workers, hunters, etc.) even require regular visits to forested regions. While *A. inermis* does not select humans as hosts often, the presence of this species in popular recreational areas increases the likelihood of incidental human contact, raising the risk of exposure to any pathogens it may carry (Bursali et al., 2012).

In Central Europe, there is a common misconception among the general public that ticks become inactive in winter, reducing the perceived need for protective measures against these parasites during colder months. Although this idea has some basis regarding other common species, it does not apply to winter ticks, as adult ticks of this species actively seek hosts throughout the winter (Otranto et al., 2017; Kahl and Gray, 2023). This misconception may lead to a false sense of security among those visiting nature in the colder months, further emphasizing the importance of monitoring *A. inermis* and clarifying its role as a potential vector.

Given the increasing impact of climate change and human-induced environmental shifts, proactive monitoring of tick populations is consistent with the DAMA protocol - Document, Assess, Monitor, Act - a preventive approach designed to address the growing threat of emerging infectious diseases (Hoberg, 2022). This protocol encourages a proactive way of tracking pathogens within changing ecological conditions, where shifts in host-pathogen relationships can catalyze the spread of new infections. In the case of *A. inermis*, monitoring its role as a vector and the potential pathogens it carries can help anticipate the risks this tick may pose to both human and animal health (Hoberg, 2022). Therefore, to obtain information about the pathogen association of this species, in our present study, we screened for pathogens carried by one *A. inermis* population using the reverse line blot (RLB) hybridization assay.

## 2 Materials and methods

### 2.1 Collection of ticks

**Fig. 1:**
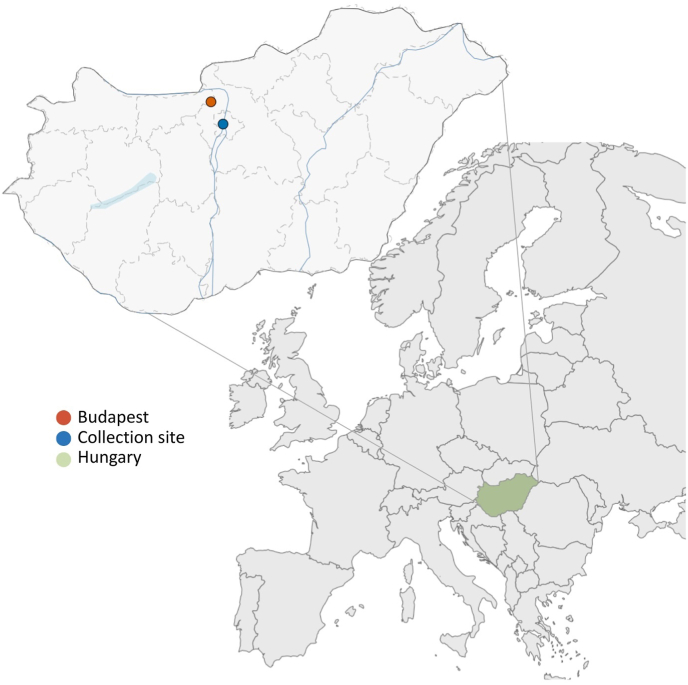
The *A. inermis* collection site (red dot) at the border of Pilisszentkereszt village in the Pilis mountain region, not far from the capital of the country, Budapest (blue dot). The location of Hungary within Europe is marked in green. Maps were produced in R using the *sf* package (Pebesma, 2018) and visualized with *ggplot2* (Wickham, 2016).

**Fig. 2:**
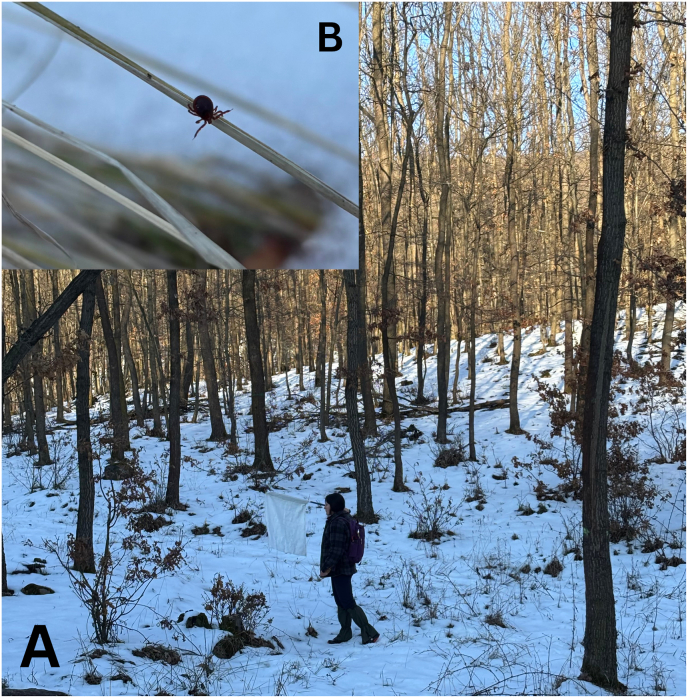
A part of the tick collection site (**A**) and an *A. inermis* specimen collected in winter at this location (**B**).

The ticks examined were collected from a forested area near Pilisszentkereszt (47°41′56.5"N, 18°54′27.8"E) in the Pilis Mountains. This location is approximately 30 km northwest of Budapest, the capital of Hungary (Fig. 1). The collection site is situated in a region that attracts many hikers and tourists due to its extensive trail network. This forest consists of a mix of deciduous trees, with a stream running through it, as well as smaller ponds and marshy regions (Fig. 2). The study area has a variety of hills, with an elevation of approximately 570 m above sea level. According to observations by Pilisi Parkerdő, the forestry company responsible for the area, the broader location is home to red deer, fallow deer, roe deer, wild boars, mouflons, hedgehogs, voles, and other small mammals. Apart from *A*. *inermis*, other species can also be found in this area, notably *Ixodes ricinus*, *H*. *concinna*, and, rarely, *Dermacentor marginatus* and *Dermacentor reticulatus* (Gábor Földvári, unpublished data).

We collected ticks using the flagging method, conducted once a month, repeatedly following the same pre-determined route. Due to the habitat heterogeneity, the sampling session was not based on distance, but on time, each collection lasted 1.5 to 2 h. During collection, ticks were stored in tubes containing 70% ethanol. We examined ticks from seven months of collection, specifically: October-December 2022, January 2023, and October-December 2023.

A total of 208 adult *A. inermis* ticks were analyzed, including 123 females and 85 males. Specimens were identified using a Nikon SMZ800N stereomicroscope (Nikon Corporation, Yokohama, Japan) based on the identification keys by Estrada-Peña et al. (2018) and Hillyard (1996).

### 2.2 DNA extraction from ticks

The DNA of all collected ticks was extracted using the DNeasy Blood and Tissue Kit (Qiagen, Hilden, Germany) as previously described (Schötta et al., 2023). Briefly, ticks were washed in 70% ethanol, air-dried completely, and cut into halves using a singular sterile surgical blade per tick. The lysis step was performed by incubation at 56 °C for between 3 h and overnight in 180 μl ATL buffer and 20 μl proteinase K. The following DNA extraction steps were performed according to the manufacturer’s protocol. DNA samples were stored at −20 °C.

### 2.3 PCR-reverse line blot analysis

Genus-specific PCR amplifications were conducted to target the 16S rRNA gene of *Anaplasma*, *Ehrlichia* (including “*Candidatus* Neoehrlichia mikurensis”), the 18S rRNA gene of *Babesia* and *Theileria*, the 5S-23S intergenic spacer (IGS) of *Borrelia burgdorferi* (*sensu lato*), and both the 16S rRNA gene and the 23S-5S IGS of *Rickettsia* spp. The reverse primers were labelled with 5’ biotin (Kong and Gilbert, 2006; Schötta et al., 2017). The PCR reaction mix contained 5 μl of Phire reaction buffer, 200 nmol/l of each dNTP (Solis BioDyne, Tartu, Estonia), 400 nmol/l of each primer per specific primer pair, 0.125 units of Phire Hot Start II DNA Polymerase (Thermo Scientific, Vienna, Austria), 2.5 μl of template DNA, and PCR-grade water (Sigma-Aldrich, Vienna, Austria) up to a final volume of 25 μl. To amplify the specific regions, touch-down PCR programs were used as previously described (Wijnveld et al., 2016). The reverse line blot assay was carried out as previously described (Nijhof et al., 2005).

The resulting PCR products from each tick were pooled individually in 2× SSPE buffer (Thermo Scientific, Vienna, Austria), which contained 0.3M NaCl, 0.02M NaH_2_PO_4_, 0.002M EDTA, and 0.1% sodium dodecyl sulfate (SDS, Applichem, Darmstadt, Germany). Following this, the combined PCR products were denatured by incubation at 100 °C and rapidly cooled on ice. The resulting single-stranded PCR products were hybridized to oligonucleotide probes covalently bound to a nylon membrane (Biodyne C, Pall Laboratories, Crailsheim, Germany). These probes consisted of genus-specific (catch-all) and species-specific oligonucleotide probes (Eurofins Genomics GmbH, Ebersberg, Germany) designed for the detection of various microorganisms. The specific probes used are detailed in Schötta et al. (2017). To enable visualization, the membrane was incubated with a 1:10,000 dilution of horseradish peroxidase-conjugated streptavidin (Roche Diagnostics GmbH, Mannheim, Germany). Detection of the hybridized PCR products was performed using the Pierce ECL Western blotting substrate (Thermo Fisher Scientific, Vienna, Austria) and the iBright CL750 Imaging System (Thermo Scientific, Vienna, Austria). For PCR quality control, DNA extracts from bacterial cultures or confirmed-positive tick samples were included. Blotting quality was monitored by using pooled biotinylated PCR amplicons from confirmed-positive samples.

### 2.4 Sanger sequencing

For confirmation, or when catch-all-only signals were observed, additional Sanger sequencing was performed. In the case of *Rickettsia*, in a number of ticks, catch-all-only signals were observed, or two species were detected simultaneously. As *Rickettsia* species are known to out-compete one another, the signals were presumed to arise from cross-reactivity and were further analyzed. Of all signals selected for further sequencing, the specific RLB-PCR was repeated without biotinylated primers. Resulting amplicons were purified from agarose gel electrophoresis using the QIAquick Gel Extraction Kit (Qiagen). The DNA fragments were sent for bidirectional sequencing to Microsynth Austria GmbH. Consensus sequences were created using Geneious Prime (https://www.geneious.com/), and the resulting sequences were analyzed using NCBI BLAST (https://blast.ncbi.nlm.nih.gov/Blast.cgi).

## 3 Results

Out of the 208 *A. inermis* collected, 150 (72.1%) tested positive for at least one pathogen, while 58 samples (27.9%) were negative for all tested agents.

The most common pathogen group identified was *Rickettsia* spp., detected using multiple species-specific probes. *Rickettsia helvetica* was the most prevalent, detected in 53 of 208 samples (25.5%), followed by *Rickettsia* spp*.* in 27 of 208 samples (13.0%), *R. massiliae* in 24 samples (11.5%), and *R. raoultii* in 11 samples (5.3%). *Rickettsia* spp. catch-all signals, and double signals that were suspected to be cross-reactivity signals, were further analyzed using Sanger sequencing. The obtained rickettsial sequences were identical to one another, and the resulting 16S rRNA consensus sequence (257 bp) from two tick specimens presented a similarity of 99.2% to *Rickettsia tillamookensis* (GenBank: CP060138.2) and *Rickettsia rhipicephali* (GenBank: CP003342.1). The consensus sequence was submitted to the GenBank database under the following name and accession number: Uncultured *Rickettsia* sp. clone Ai-2022-F9 16S ribosomal RNA gene, PX710097.

The second most common pathogen group was *Babesia* spp., with *Babesia microti* detected in 40 samples (19.2%), which occurred together with *Babesia venatorum* in one sample (0.5%)*.* Additional Sanger sequencing also revealed the presence of *Babesia crassa* (255 bp consensus sequence 100% identical to PX992076.1) in 15 ticks (7.2%). *Borrelia burgdorferi* (*s.l*.) was detected in 34 ticks (16.3%), with *Borrelia burgdorferi* (*s.s.*) identified in 23 samples (11.1%) and *B. afzelii* in 11 samples (5.3%). “*Candidatus* N. mikurensis” was found in 31 samples (14.9%). Of the total tick samples, 82 (39.4%) carried a single pathogen, 52 (25.0%) contained two pathogens, 13 (6.3%) contained three pathogens, and 3 (1.4%) contained four pathogens (Table 1).

**Table 1:** Summary of the most frequent single, double, triple, and quadruple infection combinations detected in *Alloceraea inermi*s ticks collected in Hungary.

Microorganism	No. of ticks (%)
**Single infections**	82 (39.4)
*Rickettsia helvetica*	21 (10.1)
*Rickettsia* spp.	17 (8.2)
*Babesia microti*	13 (6.3)
*Rickettsia massiliae*	12 (5.8)
*Rickettsia raoultii*	8 (3.8)
*Borrelia burgdorferi* (*s*.*s*.)	6 (2.9)
*Babesia crassa*	3 (1.4)
“*Candidatus* Neoehrlichia mikurensis”	2 (1.0)
**Double infections**	52 (25.0)
*Rickettsia helvetica +* “*Candidatus* Neoehrlichia mikurensis”	7 (3.4)
*Rickettsia helvetica + Babesia crassa*	7 (3.4)
*Rickettsia helvetica + Babesia microti*	6 (2.9)
*Rickettsia helvetica + Borrelia burgdorferi* (*s*.*s*.)	6 (2.9)
*Rickettsia massiliae + Babesia microti*	5 (2.4)
*Rickettsia massiliae +* “*Candidatus* Neoehrlichia mikurensis”	3 (1.4)
*Rickettsia* spp*. +* “*Candidatus* Neoehrlichia mikurensis”	3 (1.4)
*Babesia microti + Borrelia afzelii*	2 (1.0)
“*Candidatus* Neoehrlichia mikurensis” *+ Babesia microti*	2 (1.0)
*Babesia microti + Borrelia burgdorferi* (*s*.*s*.)	2 (1.0)
*Rickettsia helvetica + Borrelia afzelii*	1 (0.5)
*Rickettsia* spp*. + Babesia crassa*	1 (0.5)
*Rickettsia* spp*. + Borrelia afzelii*	1 (0.5)
*Rickettsia* spp*. + Babesia microti*	1 (0.5)
“*Candidatus* Neoehrlichia mikurensis” *+ Babesia crassa*	1 (0.5)
*Rickettsia raoultii +* “*Candidatus* Neoehrlichia mikurensis”	1 (0.5)
*Rickettsia massiliae + Borrelia burgdorferi* (*s*.*s*.)	1 (0.5)
*Borrelia afzelii + Babesia crassa*	1 (0.5)
*Rickettsia raoultii + Borrelia burgdorferi* (*s*.*s*.)	1 (0.5)
**Triple infections**	13 (6.3)
*Rickettsia* spp*. +* “*Candidatus* Neoehrlichia mikurensis” *+ Babesia microti*	2 (1.0)
*Rickettsia massiliae +* “*Candidatus* Neoehrlichia mikurensis” *+ Babesia microti*	2 (1.0)
*Rickettsia helvetica +* “*Candidatus* Neoehrlichia mikurensis” *+ Borrelia burgdorferi* (*s*.*s*.)	2 (1.0)
*Rickettsia raoultii +* “*Candidatus* Neoehrlichia mikurensis” *+ Babesia microti*	1 (0.5)
*Rickettsia helvetica +* “*Candidatus* Neoehrlichia mikurensis” *+ Babesia microti*	1 (0.5)
*Babesia microti + Babesia venatorum + Borrelia afzelii*	1 (0.5)
*Rickettsia* spp*. + Borrelia burgdorferi* (*s*.*s*.) *+ Borrelia afzelii*	1 (0.5)
“*Candidatus* Neoehrlichia mikurensis” *+ Borrelia burgdorferi* (*s*.*s*.) *+ Babesia crassa*	1 (0.5)
*Rickettsia helvetica +* “*Candidatus* Neoehrlichia mikurensis” *+ Borrelia afzelii*	1 (0.5)
*Rickettsia massiliae +* “*Candidatus* Neoehrlichia mikurensis” *+ Borrelia burgdorferi* (*s*.*s*.)	1 (0.5)
**Quadruple infections**	3 (1.4)
*Rickettsia helvetica + Babesia microti + Borrelia burgdorferi* (*s*.*s*.) *+ Borrelia afzelii*	1 (0.5)
*Rickettsia massiliae +* “*Candidatus* Neoehrlichia mikurensis” *+ Babesia microti + Borrelia afzelii*	1 (0.5)
*Rickettsia* spp*. + Borrelia burgdorferi* (*s*.*s*.) + *Borrelia afzelii + Babesia crassa*	1 (0.5)

## 4 Discussion

In the present study, we screened the tick-borne pathogen associations of 208 unfed *A*. *inermis*. Based on our findings, these ticks can harbor a diverse set of pathogens, including several of considerable relevance to both human and veterinary health.

Notably, a broad spectrum of *Rickettsia* species was detected, with *R. helvetica* in 25.5% of the ticks, followed by *Rickettsia* spp. in 13.0% of the ticks, *R. massiliae* in 12.0% of the ticks, and *R. raoultii* in 5.3% of the ticks. While several *Rickettsia* species are considered non-pathogenic to humans, infections often present as a febrile illness with fever, headache, and skin lesions (Faccini-Martínez et al., 2014). The range of *Rickettsia* spp. observed in the present study shows both similarities and differences compared with previously published data for other tick species from Central Europe. In Slovakia, screening of *I*. *ricinus* ticks detected *Rickettsia* DNA in approximately 9% of samples, with *R. helvetica* being the most prevalent (77 of 87) in the positive ticks, followed by *R. monacensis* in 8 ticks (Špitalská et al., 2014). Similarly, previous studies in Hungary identified *R. helvetica* and *R. monacensis* as the predominant rickettsial species in *I. ricinus*, with *R. helvetica* prevalence as high as 45% in female ticks (Szekeres et al., 2016). Beyond *Ixodes* spp., a survey conducted in south-eastern Slovakia investigated the ticks *Dermacentor reticulatus* and *A*. *inermis*, and detected *Rickettsia* spp. DNA in 23.72% (*n* = 59) of *D. reticulatus* and 16.76% (*n* = 173) of *A. inermis* samples. Subsequent sequencing confirmed the presence of *R. raoultii* in 13 ticks belonging to both species (Ouarti et al., 2022).

The second most prevalent pathogen group in our study was *Babesia* spp*.* in 26.9% of winter ticks. *Babesia microti* was among the most frequently detected pathogens in our samples, occurring in 19.2% of *A*. *inermis*, while *B*. *venatorum* was detected in one sample together with *B. microti* (0.5%). Additional sequencing also revealed the presence of *B*. *crassa* in 7.2% of ticks examined. In comparison, studies in Central Europe showed relatively low *Babesia* spp. prevalence, for example, 1.5% in *I. ricinus* and 6.6% in *H*. *concinna* in south-west Slovakia (Hamšíková et al., 2016). Similarly, in eastern Poland, *B. microti* was detected in 1.5%, and *B. venatorum* in 1.2% of questing *I. ricinus* ticks (Sawczyn-Domańska et al., 2023). These results, compared to our data, suggest a locally higher circulation of *Babesia* spp. in the study region and a possible role of *A. inermis* in the maintenance of these protozoans.

We detected *B. burgdorferi* (*s.s.*) in 11.1% and *Borrelia afzelii* in 5.3% of the ticks analyzed. In comparison, a long-term study in Slovakia detected *B. burgdorferi* (*s.l.*) species with an overall infection prevalence of 18.8% in *I. ricinus*. Among this complex, *B. afzelii* was the most frequently identified genospecies (37.1%), while *B. burgdorferi* (*s.s.*) accounted for 4.1% of all positive ticks (Rusňáková Tarageľová et al., 2024). Similarly, in Hungary, seasonal monitoring of *I*. *ricinus* revealed lower average prevalences, with *B. burgdorferi* (*s.s.*) detected in 1.2% and *B. afzelii* in 0.11% of questing ticks (Egyed et al., 2012).

“*Candidatus* N. mikurensis” was detected in 14.9% of the ticks. In comparison, studies in Central Europe reported usually lower prevalence in questing *I. ricinus* ticks, with a Slovak survey finding overall infection rates ranging between 1.0% and 2.3% across multiple habitats. Additionally, a study from the Czech Republic showed an overall prevalence of 4.4% based on pooled PCR samples (Svitálková et al., 2016; Ondruš et al., 2020). In Hungary, “*Candidatus* Neoehrlichia mikurensis” was also detected in questing *I*. *ricinus* ticks, with a prevalence of 8.8% (Szekeres et al., 2015). The notably higher prevalence in our *A. inermis* samples compared with that of *I. ricinus* in neighboring countries highlights its possible role in the ecology of “*Candidatus* N. mikurensis”. This possibility calls for further investigation, given the pathogen’s recognized relevance to human health, as it can cause inflammatory disease in susceptible individuals.

Co-infections were common (32.7%) among the ticks analyzed, with many individuals harboring two or more pathogens simultaneously. *Rickettsia* spp., particularly *R. helvetica*, *R. massiliae*, *Rickettsia* spp., and *R. raoultii*, were present across almost all types of co-infections. *Borrelia* spp., mainly *B. burgdorferi* (*s*.*s*.) and *B. afzelii*, were less common, while “*Candidatus* N. mikurensis” was rarely detected alone but frequently co-occurred with *Rickettsia* spp. or *Babesia* spp. Some pathogens were detected in very few ticks, and certain co-infection combinations were extremely rare, so no strong conclusions can be drawn about these cases. Co-infections may arise through transstadial transmission and, for certain pathogens, transovarial transmission, as well as from co-feeding on the same host or interrupted feeding on multiple hosts during a single life stage. Co-infections are important, as they increase the risk of simultaneous transmission of various pathogens to hosts, potentially complicating both diagnosis and disease outcomes (Philippe et al., 2024).

Comparing co-infections detected in winter ticks with other tick species is challenging, as only a few studies have focused on *A. inermis*, and different pathogens have often been targeted in screenings of other tick species. Nevertheless, some reference can be made to previous research from the Netherlands, where 37.0% of *I. ricinus* ticks were infected with at least one pathogen, and only 6.3% carried more than one (Coipan et al., 2013). In a Romanian study, co-infections occurred in 27.4% of ticks across four species (*A. inermis* was not included), and based on previous studies from European countries, co-infection prevalence in ticks has been reported to range from 3.2% to 45.0% (Borşan et al., 2021).

In addition to detecting widespread and emerging pathogens, our findings show that *A*. *inermis* is an understudied tick species that may harbor a previously uncharacterized *Rickettsia* species, urging further investigation. For a *Rickettsia* isolate to be considered as one of the known species, it needs a similarity in the variable region of the 16S rRNA gene higher than 99.8% (Fournier et al., 2003). Although the consensus sequence has a size of 257 bp (GenBank: PX710097), and the discriminatory power can be considered insufficient, as it presents a lower similarity (99.2%) with the closest related species, *R. tillamookensis* (GenBank: CP060138.2) and *R. rhipicephali* (GenBank: CP003342.1)*,* it may suggest that our isolates are of an uncharacterized species. Both of these species are usually associated with different tick species, with *R. tillamookensis* being associated with *Ixodes pacificus* (Gauthier et al., 2021) and *R. rhipicephali* with *Rhipicephalus sanguineus* and occasionally with species of *Amblyomma*, *Dermacentor*, and *Haemaphysalis* (Labruna et al., 2007; Ortega-Morales et al., 2019; Francis et al., 2020; Duncan et al., 2021; Chatanga et al., 2025). Additionally, both of these *Rickettsia* species are associated with ticks located in the American continent, both North (Francis et al., 2020; Duncan et al., 2021; Gauthier et al., 2021), Central (Ortega-Morales et al., 2019), and South America (Labruna et al., 2007). Future work should focus on the cultivation and characterization of our isolates to confirm whether it is an uncharacterized species.

*Alloceraea inermis* has not been considered an important vector species because it rarely parasitizes humans and has therefore received limited attention. However, our results indicate that this tick might be associated with a wide range of pathogens from multiple genera, often surpassing prevalences in *I. ricinus*. During winter, *A. inermis* may become locally dominant in forested habitats, potentially contributing to pathogen persistence in these environments, especially given its ecological overlap with other tick species. Moreover, because shared animal hosts are also parasitized by ticks that more frequently bite humans, *A. inermis* may contribute to the circulation and maintenance of human pathogens, emphasizing the importance of investigating its pathogen associations.

The aforementioned brief feeding duration of *A. inermis* larvae and nymphs, which complete engorgement within a few hours, poses a significant challenge for their detection on vertebrate hosts. Because of this, incidental encounters by the public during recreational activities or on companion animals could likely go unnoticed. This special behavior may also contribute to the underestimation of the prevalence of larvae and nymphs of this species in ecological surveys and field studies, as these, unlike adult specimens, cannot be collected *via* flagging or dragging.

In accordance with the DAMA (Document, Assess, Monitor, Act) protocol, it is essential not only to document and assess this species’ vector potential but also to plan the next steps. Future studies should include expanded monitoring of winter tick populations across different locations, alongside investigations into how its unique and rapid life cycle influences its role in pathogen transmission.

## 5 Conclusions

Our findings emphasize the significant, yet understudied, pathogen associations of the winter tick *A. inermis*. The high prevalence of pathogens underscores the need for greater attention to this tick species, especially during the colder months when tick activity is often underestimated. In addition, during the winter season, people are less likely to expect tick exposure and are less vigilant; therefore, winter tick bites are more likely to remain unnoticed. The detection of a high prevalence of several pathogens and the uncharacterized *Rickettsia* species (GenBank: PX710097) highlights the need for further research into the diversity of microorganisms they might carry. These results suggest that *A. inermis* could pose a more substantial public and veterinary health risk than previously recognized.

## Ethical approval

Not applicable.

## CRediT authorship contribution statement

**Éva S. Szabó:** Conceptualization, Formal analysis, Investigation, Methodology, Project administration, Validation, Visualization, Writing - original draft, Writing - review & editing. **Margarida Ruivo:** Formal analysis, Investigation, Methodology, Project administration, Validation, Visualization, Writing - original draft, Writing - review & editing, Supervision. **Domonkos Adorján Köves:** Investigation, Writing - review and editing. **Máté Miklós:** Investigation, Writing - review and editing. **Flóra Kulin:** Investigation. **Michiel Wijnveld:** Conceptualization, Formal analysis, Funding acquisition, Methodology, Resources, Supervision, Writing - review & editing. **Gábor Földvári:** Conceptualization, Formal analysis, Funding acquisition, Methodology, Resources, Supervision, Writing - original draft, Writing - review & editing.

## Statement on the use of AI-assisted technologies

During the preparation of this work, the authors used Grammarly in order to check grammar. After using this tool, the authors reviewed and edited the content as needed and take full responsibility for the content of the published article.

## Funding

This research was supported by the National Research, Development and Innovation Office in Hungary (RRF-2.3.1-21-2022-00006 and 152906), Pipeline for Rapid Diagnostics of Emergency Transboundary Infectious Diseases (PREPARE-TID, 101137132) and in part by the Austrian Science Fund (FWF, Grant DOI: 10.55776/P33867).

## Declaration of competing interests

The authors declare that they have no known competing financial interests or personal relationships that could have appeared to influence the work reported in this paper.

## Data Availability

All data generated or analyzed during the study are included in this published article. The newly generated sequence for “Uncultured Rickettsia sp. clone Ai-2022-F9” 16S rRNA gene was submitted to the GenBank database under the accession number PX710097.
